# Comparative Analysis of AI Models for Atypical Pigmented Facial Lesion Diagnosis

**DOI:** 10.3390/bioengineering11101036

**Published:** 2024-10-17

**Authors:** Alessandra Cartocci, Alessio Luschi, Linda Tognetti, Elisa Cinotti, Francesca Farnetani, Aimilios Lallas, John Paoli, Caterina Longo, Elvira Moscarella, Danica Tiodorovic, Ignazio Stanganelli, Mariano Suppa, Emi Dika, Iris Zalaudek, Maria Antonietta Pizzichetta, Jean Luc Perrot, Gabriele Cevenini, Ernesto Iadanza, Giovanni Rubegni, Harald Kittler, Philipp Tschandl, Pietro Rubegni

**Affiliations:** 1 Dermatology Unit, Department of Medicine, Surgery and Neuroscience, University of Siena, 53100 Siena, Italy; 2 Department of Medical Biotechnologies, University of Siena, 53100 Siena, Italy; 3 Department of Dermatology, University of Modena and Reggio Emilia, 41121 Modena, Italy; 4 First Department of Dermatology, Aristotle University, 541 24 Thessaloniki, Greece; 5 Department of Dermatology and Venereology, Institute of Clinical Sciences, Sahlgrenska Academy, University of Gothenburg, Sahlgrenska University Hospital, 413 45 Gothenburg, Sweden; 6 Department of Dermatology and Venereology, Region Vastra Gotaland, Sahlgrenska University Hospital, 413 45 Gothenburg, Sweden; 7 Centro Oncologico ad Alta Tecnologia Diagnostica, Azienda Unità Sanitaria Locale, IRCCS di Reggio Emilia, 42123 Reggio Emilia, Italy; 8 Dermatology Unit, University of Campania Luigi Vanvitelli, 80138 Naples, Italy; 9 Dermatology Clinic, Medical Faculty, University of Nis, 18000 Nis, Serbia; 10 Skin Cancer Unit, Scientific Institute of Romagna for the Study of Cancer, Istituto di Ricovero e Cura a Carattere Scientifico, Istituto Romagnolo per lo Studio dei Tumori, 47014 Meldola, Italy; 11 Department of Dermatology, University of Parma, 43121 Parma, Italy; 12 Department of Dermatology, Hôpital Erasme, Université Libre de Bruxelles, 1050 Brussels, Belgium; 13 Groupe d’Imagerie Cutanée Non-Invasive, Société Française de Dermatologie, 75009 Paris, France; 14 Department of Dermatology, Institut Jules Bordet, 1070 Brussels, Belgium; 15 Oncologic Dermatology Unit, Istituto di Ricovero e Cura a Carattere Scientifico, Azienda Ospedaliero Universitaria Bologna, 40138 Bologna, Italy; emi.dika3@unibo.it; 16 Department of Medical and Surgical Sciences, University of Bologna, 40126 Bologna, Italy; 17 Dermatology Clinic, Ospedale di Trieste, 34141 Trieste, Italy; 18 Department of Medical Oncology, Centro di Riferimento Oncologico di Aviano, 33081 Aviano, Italy; 19 Dermatology Unit, University Hospital of St-Etienne, 42270 Saint Etienne, France; 20 Ophthalmology Unit, Department of Medicine, Surgery and Neuroscience, University of Siena, 53100 Siena, Italy; 21 Department of Dermatology, Medical University of Vienna, 1090 Vienna, Austria

**Keywords:** machine learning, deep learning, dermatoscopy, convolutional neural network, logistic regression, skin cancer, pigmented facial lesions, iDScore

## Abstract

Diagnosing atypical pigmented facial lesions (aPFLs) is a challenging topic for dermatologists. Accurate diagnosis of these lesions is crucial for effective patient management, especially in dermatology, where visual assessment plays a central role. Incorrect diagnoses can result in mismanagement, delays in appropriate interventions, and potential harm. AI, however, holds the potential to enhance diagnostic accuracy and provide reliable support to clinicians. This work aimed to evaluate and compare the effectiveness of machine learning (logistic regression of lesion features and patient metadata) and deep learning (CNN analysis of images) models in dermoscopy diagnosis and the management of aPFLs. This study involved the analysis of 1197 dermoscopic images of facial lesions excised due to suspicious and histologically confirmed malignancy, classified into seven classes (lentigo maligna—LM; lentigo maligna melanoma—LMM; atypical nevi—AN; pigmented actinic keratosis—PAK; solar lentigo—SL; seborrheic keratosis—SK; and seborrheic lichenoid keratosis—SLK). Image samples were collected through the Integrated Dermoscopy Score (iDScore) project. The statistical analysis of the dataset shows that the patients mean age was 65.5 ± 14.2, and the gender was equally distributed (580 males—48.5%; 617 females—51.5%). A total of 41.7% of the sample constituted malignant lesions (LM and LMM). Meanwhile, the benign lesions were mainly PAK (19.3%), followed by SL (22.2%), AN (10.4%), SK (4.0%), and SLK (2.3%). The lesions were mainly localised in the cheek and nose areas. A stratified analysis of the assessment provided by the enrolled dermatologists was also performed, resulting in 2445 evaluations of the 1197 images (2.1 evaluations per image on average). The physicians demonstrated higher accuracy in differentiating between malignant and benign lesions (71.2%) than in distinguishing between the seven specific diagnoses across all the images (42.9%). The logistic regression model obtained a precision of 39.1%, a sensitivity of 100%, a specificity of 33.9%, and an accuracy of 53.6% on the test set, while the CNN model showed lower sensitivity (58.2%) and higher precision (47.0%), specificity (90.8%), and accuracy (59.5%) for melanoma diagnosis. This research demonstrates how AI can enhance the diagnostic accuracy in complex dermatological cases like aPFLs by integrating AI models with clinical data and evaluating different diagnostic approaches, paving the way for more precise and scalable AI applications in dermatology, showing their critical role in improving patient management and the outcomes in dermatology.

## 1. Introduction

Melanoma is a malignant tumour originating from melanocytes, the cells responsible for producing melanin, the pigment that gives skin its colour. While it is curable with excision in the early stages, it is an aggressive form of skin cancer in more advanced stages. The incidence of melanoma has risen globally. A new study from the International Agency for Research on Cancer (IARC) and its partners predicted that the number of new cases of cutaneous melanoma per year will increase by more than 50% from 2020 to 2040, raising from 325,000 to 510,000 new cases, with a 68% increase in deaths (from 57,000 to 96,000 deaths per year) [1]. In the United States alone, it is projected that about 100,640 new cases of invasive melanoma will be diagnosed, leading to approximately 8290 deaths [2]. These data highlight the continuing burden of melanoma [3] and underscore the importance of ongoing research and advances in melanoma treatment. There is ongoing discussion about the risk of overdiagnosis in the context of pigmented skin lesions [4]. Facial melanomas, particularly lentigo maligna (LM) and lentigo maligna melanoma (LMM), are often associated with chronic exposure to ultraviolet (UV) light. LM is an in situ melanoma characterised by atypical melanocytes along the basal layer of the epidermis. It typically affects elderly individuals and is most commonly found on the face and neck. When LM invades the dermis, it becomes LMM, a more aggressive form of melanoma [5]. Atypical pigmented facial lesions (aPFLs) refer to unusual or abnormal spots or areas of discolouration on the face that are darker than the surrounding skin. These lesions can vary in colour, size, shape, and texture and may not fit the typical characteristics of common skin conditions. aPFLs pose a unique challenge for dermatologists due to their complex visual characteristics and the high stakes of an accurate diagnosis. Diagnosing facial melanoma is challenging due to its atypical presentation and the presence of benign lesions that can mimic its appearance. The introduction of dermoscopy—i.e., the examination of a skin lesion at high magnification using transillumination to visualise its subtle features—has been a watershed moment in the early detection of malignant melanoma [6]. However, dermoscopic-based diagnosis is operator-dependent and requires extensive personal training [7]. Moreover, a misdiagnosis could result in unneeded surgery or the incorrect treatment [8]. Epidemiological studies that have assessed skin screening have found that it leads to increased detection of tumours, which suggests a high degree of overdiagnosis [9].

Artificial intelligence (AI) has emerged as a transformative technology in numerous fields, including medicine. AI offers tools that enhance diagnostic accuracy, treatment planning, and patient management. Within AI, machine learning (ML) and deep learning (DL) are capable of processing vast amounts of data, identifying patterns, and enhancing the diagnostic accuracy and more objective decision-making in healthcare by reducing misdiagnoses and overdiagnosis [10]. These capabilities are particularly valuable in fields that rely heavily on image analysis, such as radiology, pathology, and dermatology [11]. ML encompasses a variety of algorithms that enable computers to learn from data. These algorithms can be broadly categorised into supervised learning and unsupervised learning. Supervised learning (e.g., logistic regression—LR; linear regression; decision tree; random forest; and support vector machines), which involves training a model on a labelled dataset, is particularly useful for classification tasks such as diagnosing diseases based on medical images [12]. Unsupervised learning (e.g., hierarchical clustering, k-means clustering), which identifies patterns in data without labelled outcomes, is often used for clustering and dimensionality reduction [13]. On the other hand, DL involves neural networks with multiple layers that can learn complex data representations. Convolutional neural networks (CNNs) are a type of DL model specifically designed for image analysis. They use convolutional layers to automatically detect features such as edges, textures, and shapes within images, making them particularly effective for tasks such as image classification and object detection, outperforming the traditional methods [14]. CNN-based decision support systems are proven to be efficient tools in dermatology, as they can help reduce the ratio of inappropriate excisions [8]. However, CNNs may exhibit poor performance on skin lesion segmentation tests due to a lack of knowledge of the long-range spatial linkages in skin lesion images. AI vision models based on the Transformer deep learning architecture introduced in 2017 [15], such as Vision Transformers (ViTs) [16], can address this constraint [17]. Moreover, recent studies have proposed modified DL models [18] and multimodal AI systems [19] for improving skin cancer classifications. Multimodal Large Language Models (MLLMs) may have a strong impact on visual medical specialities such as dermatology. Specifically, they may have the capability to free up physicians’ time and allow them to focus on more complex cases and critical patient care by learning simultaneously from images and words to tackle several tasks, including image recognition, answering visual questions, understanding documents, and image captioning.

This study aims to compare a traditional ML approach with advanced DL techniques by evaluating and comparing the effectiveness of two AI-based models—a logistic regression-based scoring model and a CNN model—in the diagnosis of aPFLs. The training dataset employed for both models is made up of 1197 dermoscopic images of facial lesions excised due to suspicious and histologically confirmed malignancy collected within the EU Integrated Dermoscopy Score (iDScore) project. The CNN-based model will be trained only on the raw images. The key advantage of using a CNN for this task is that it can automatically learn the important features without the need to incorporate other features (such as patient metadata) to use as inputs to the model, as the CNN will learn these features automatically from the data, providing an objective way to evaluate aPFLs. Moreover, recent studies have demonstrated how the integration of dermoscopic images with metadata does not reflect a substantial increase in the model performance [8,20]. On the other hand, the LR-based model will also incorporate patient metadata and dermoscopic features identified by dermatologists, whose assessment is subjective and dependent on their experience as physicians.

Its ultimate scope is to provide insights about their potential and limitations in improving diagnostic accuracy and patient management.

## 2. Materials and Methods

The iDScore project is a multicentric European project, promoted by the working group of the teledermatology task force of the European Academy of Dermatology and Venereology and focused on the development of a decision support system to improve the diagnosis of difficult melanoma skin lesions [21]. The iDScore project has three different sub-projects: the iDScore-PalmoPlantar project, the iDScore-Body project, and the iDScore-Facial project. The three projects aim to study melanoma and its simulators and to create decision support systems to help dermatologists in diagnosing and distinguishing between them. The iDScore-Facial project was a retrospective multicentric study aimed at developing a diagnostic support tool for the differential diagnosis of LM [22,23]. It constituted two phases: image collection and image testing. The former consisted of the enrolment of several centres that contributed to providing dermoscopic images and other information on melanoma and non-melanoma cases (Section 2.1). The latter consisted of the evaluation of these images by European dermatologists with various levels of experience (Section 2.2).

### 2.1. Image Collection

**Figure 1:**
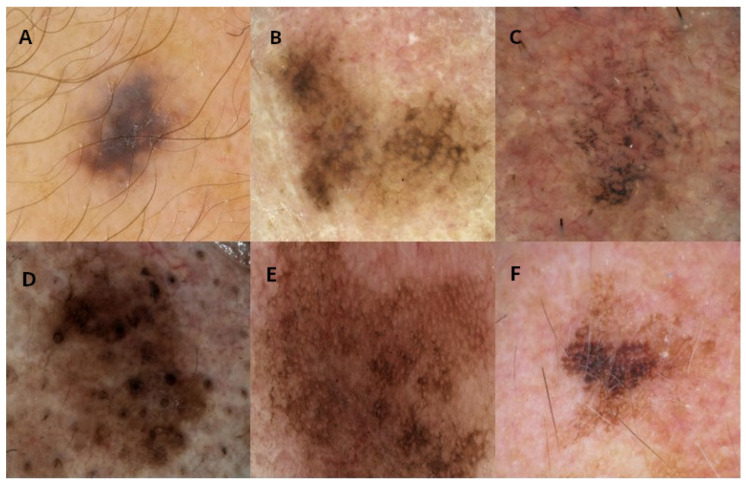
Examples of images for each diagnosis in the iDScore database. (**A**) Atypical nevi, (**B**) lentigo maligna, (**C**) pigmented actinic keratosis, (**D**) seborrheic keratosis, (**E**) seborrheic lichenoid keratosis, (**F**) solar lentigo.

This study involved a retrospective, multicentric collection of dermoscopic images from twelve European centres: the Universities of Siena (Italy), Modena (Italy), Reggio Emilia (Italy), Napoli (Italy), Bologna (Italy), Aviano (Italy), Trieste (Italy), Thessaloniki (Greece), Nis (Serbia), Saint Etienne (France), Brussels (Belgium), and Gothenburg (Sweden). Each centre provided at least 80 cases of facial skin lesions, including a minimum of 30 malignant and 50 benign lesions. Lesions on the eyelids, lips, and ears were excluded from the study. Each lesion was excised due to suspicion of malignancy and subsequently confirmed by histological examination. The dataset included two malignant diagnoses (LM and LMM) and five benign diagnoses (atypical nevi—AN; pigmented actinic keratosis—PAK; solar lentigo—SL; seborrheic keratosis—SK; and seborrheic lichenoid keratosis—SLK) (Table 1). For each lesion, a high-quality dermoscopic image (at 10× to 20× magnification, in JPEG/TIFF format, and with a resolution > 150 dpi) was provided (Figure 1). Optional clinical images were included when they were available.

The mandatory data collected for each case included the histological diagnosis, the patient’s sex, the patient’s age, and the lesion’s diameter. Optional data included the phototype, the presence of pheomelanin, whether the patient had blond hair, whether they had green/light blue eyes, history of multiple nevi, family history of melanoma, and history of sunburns before the age of 14.

### 2.2. Image Testing

**Figure 2:**
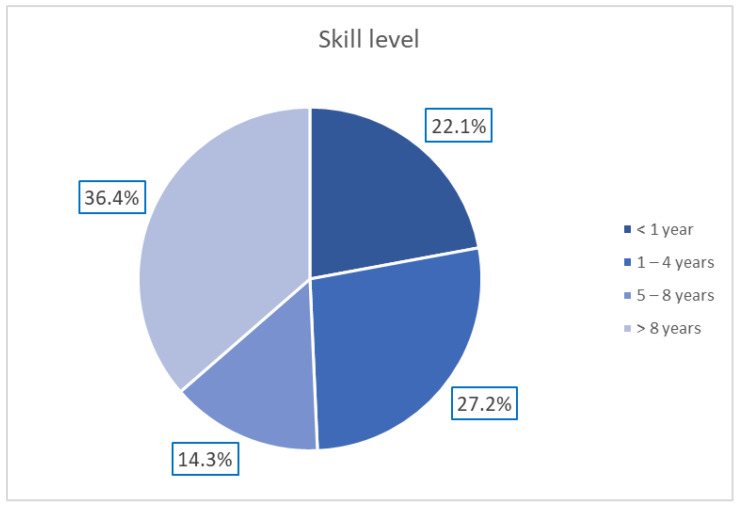
Distribution of dermatologists’ expertise in dermoscopy.

A total of 154 European dermatologists with varying levels of experience in dermoscopy participated in this study according to the protocol described by Tognetti et al. [23]. The distribution of the dermatologists’ experience is reported in Figure 2.

Each dermatologist was provided with a panel of 20 cases to evaluate. The panels were randomly assigned, ensuring that the participants did not assess images from their own centres. Each panel included 12 benign and 8 malignant cases, with no indication of the distribution provided to the participants. The evaluation process required the dermatologists to assess (as present/not present) 14 dermoscopic patterns [23] (Table 2). For each image, the physicians had to make a pattern diagnosis, rate their confidence in their diagnosis and the difficulty of each case on a 5-point Likert scale (very easy, easy, moderate, difficult, or very difficult) [24], and recommend a management plan (e.g., excision/biopsy, reflectance confocal microscopy, other non-invasive examinations, or close follow-up).

### 2.3. Model Development

Two models were developed for this study: a logistic regression-based scoring model and a CNN model. The former was developed with R v4.3.1, while the latter was developed with Python v3.8 and the PyTorch framework v2.2.0.

- The Logistic Regression Scoring Model: This model was developed using a stepwise logistic regression approach, incorporating the 14 dermoscopic patterns described in Section 2.2, the patient’s age and sex, and lesion diameter as the predictor variables. The binary outcome consisted of malignant lesions (LM + LMM) vs. benign lesions (SK + SL + SLK + PAK + AN). The stepwise procedure was a forward–backward procedure based on the Area Under the Receiver Operating Characteristic (AUROC) [25]. A variable could be added or removed only if it contributed at least 0.003 of the AUROC and was statistically significant. The model was trained and validated with a 5-fold cross-validation technique on 80% of the dataset. The best-performing model was then selected and tested on the remaining 20% of the data. The coefficients were transformed into integer scores to create a user-friendly scoring system for clinical use.
- The CNN Model: A ResNet-34 architecture [26] was employed. Other experiments with more complex models, such as ResNet-101, EfficientNet B0, and EfficientNet B1, have also been performed previously, obtaining similar or worse results. For this reason, the simplest model (ResNet-34) was chosen and presented. The pre-trained model was fine-tuned on 1197 collected images (see Section 2.1) and 743 images of facial aPFLs extracted from the International Skin Imaging Collaboration (ISIC) 2018 dataset [27]. LM and LMM diagnoses were aggregated because of their similar superficial patterns. The model was trained and validated with 5-fold cross-validation and finally tested on 111 unseen images. Data augmentation was performed on the dataset by applying geometric and colour transformations: crop (probability = 0.1), horizontal flip (probability = 0.5), vertical flip (probability = 0.5), and colour transformations (brightness, contrast, and saturation transformations with probability = 0.1). The final parameters for training the CNN model were selected after 5-fold cross-validation to optimize the performance (Table 3). An early stopping rule was also defined to manage overfitting. The training stopped if the validation loss did not decrease by at least 0.03 within 10 epochs. The final model was that with the lowest loss at the beginning of the early stopping epoch count. Cross-entropy weighted for class frequency was chosen as the loss function, the AdamW stochastic gradient descent method was chosen as the optimizer, and “reduce learning rate on plateau” was chosen as the learning rate scheduler (factor = 0.1, patience = 3, and threshold = 0.0001) [28].

## 3. Results

### 3.1. Statistical Analysis

**Figure 3:**
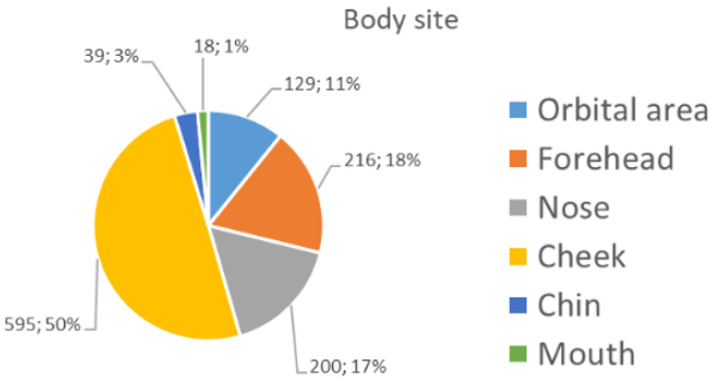
Distribution of the specific subareas of the face.

The majority (90%) of the 1197 images collected (see Section 2.1) were captured with a camera-based system or a videodermatoscope. The remaining images were collected with a smartphone-based system. The patients’ mean age (±standard deviation) was 65.5 ± 14.2, and their gender was equally distributed (580 males—48.5%; 617 females—51.5%). A total of 41.7% of the sample constituted malignant lesions (LM and LMM). Meanwhile, the benign lesions were mainly PAK (19.3%), followed by SL (22.2%), AN (10.4%), SK (4.0%), and SLK (2.3%) (Table 1). Figure 3 shows the distribution of the anatomical sites of the collected lesions. The lesions were mainly localised in the cheek and nose areas.

Since experience is a major factor influencing diagnostic accuracy and pattern recognition, a stratified analysis of the assessment provided by the enrolled dermatologists was performed. At the end of the image testing phase, 2445 evaluations of 1197 images were obtained (2.1 evaluations per image on average). Considering the assessment of case difficulty, the dermatologists found the malignant lesions were the most challenging to evaluate. Similarly, cases that were considered easier to assess coincided with SL. Regarding their diagnostic confidence, the dermatologists also felt more confident about SL, but once again, they were less confident about malignant lesions (Table 4). Histology was not known to the participants at this stage.

The physicians demonstrated higher accuracy in differentiating between malignant and benign lesions (71.2%) than in distinguishing between the seven specific diagnoses across all the images (42.9%, Table 5).

### 3.2. Logistic Regression Model Performance

The initial model constituted the 14 dermoscopic patterns (Section 2.2), the lesion’s maximum diameter, its specific location on the body, patient sex, and patient age. The age was transformed into five dichotomous variables, setting the cut-offs at 30, 40, 50, 60, and 70 years old. In the same way, the diameter was transformed into six different dichotomous variables, setting the cut-offs at 4, 8, 12, 16, 20, and 24. All of the coefficients of the logistic model could be only positive. Five-fold cross-validation was performed, and the best model was chosen. The best model is described in Table 6. It has 10 variables (diameter, age, sex, and the seven dermoscopic patterns). The coefficients of the logistic regression were standardised and then rounded to an integer. The resulting score (namely iDScore-Facial) varied from 0 to 16.

**Figure 4:**
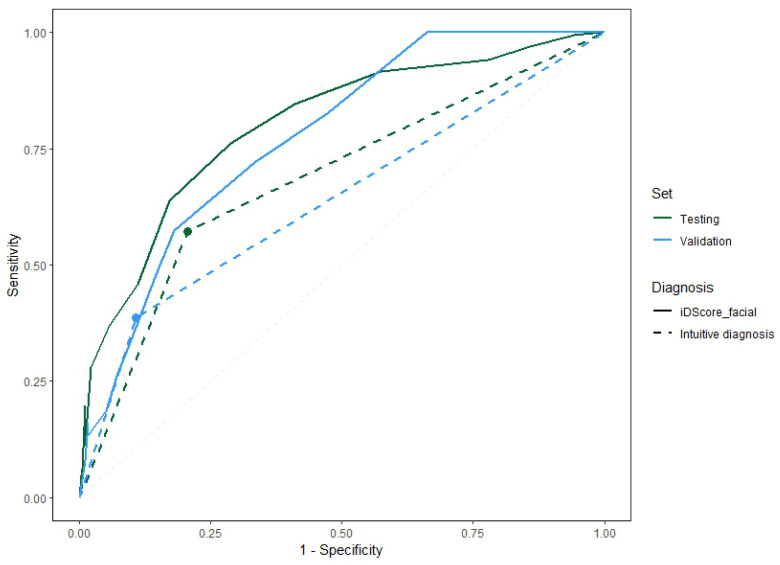
ROC curves for the model on the validation and testing samples and the pattern recognition diagnoses of the dermatologists.

Figure 4 shows the Receiver Operating Characteristic (ROC) curves of the model for the two samples comparing the performance obtained with the iDScore-aided diagnoses and with intuitive clinical diagnoses. Three ranges were then defined based on the distribution of benign and malignant cases of each score value:

- Very low (range of 0–2): Malignant lesions are rarely observed within this score range.
- Intermediate (range of 3–9): It is not possible to confidently determine whether a lesion is more likely benign or malignant in this range.
- Very high (range of 10–16): Observed lesions are highly likely to be malignant in this range.

The distribution of LM/LMM and benign cases for the model on the testing set within the three identified ranges is reported in Table 7. The cut-off value was chosen to maximize the sensitivity of the model in classifying the model diagnoses into malignant/benign cases. By choosing a value of 3, a sensitivity of 100%, a specificity of 33.9%, a precision of 39.1%, and an accuracy of 53.6% were obtained on the test set.

The values of the direct diagnosis were added to compare the model performance with the direct diagnosis performance. The difference in the area between continuous and dashed lines of the same colour shows the impact of the model in terms of its diagnostic accuracy.

### 3.3. CNN Model Performance

**Figure 5:**
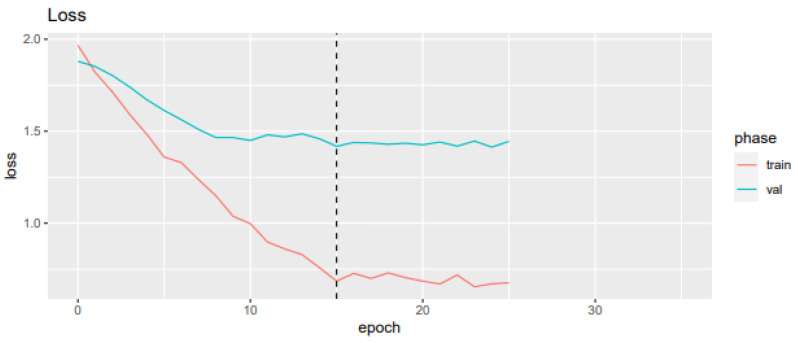
Loss value and mean recall and accuracy for each epoch in the training and validation sample.

**Figure 6:**
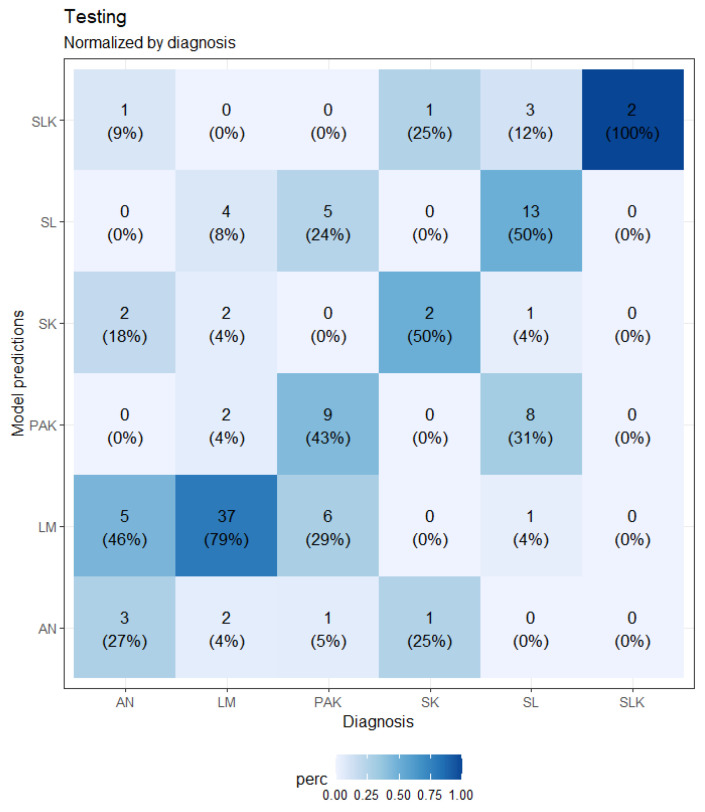
Confusion matrix of the CNN model on the testing sample.

A loss plot during training and validation for the best-performing CNN model after five-fold cross-validation is depicted in Figure 5, while a confusion matrix is shown in Figure 6.

The CNN-based model obtained a mean sensitivity of 58.2%, a specificity of 90.8%, a precision of 47.0%, and an accuracy of 59.5%. Table 8 shows the sensitivity of the model for each class on the testing samples compared to the sensitivity of the responses of the dermatologists involved.

## 4. Discussion

**Figure 7:**
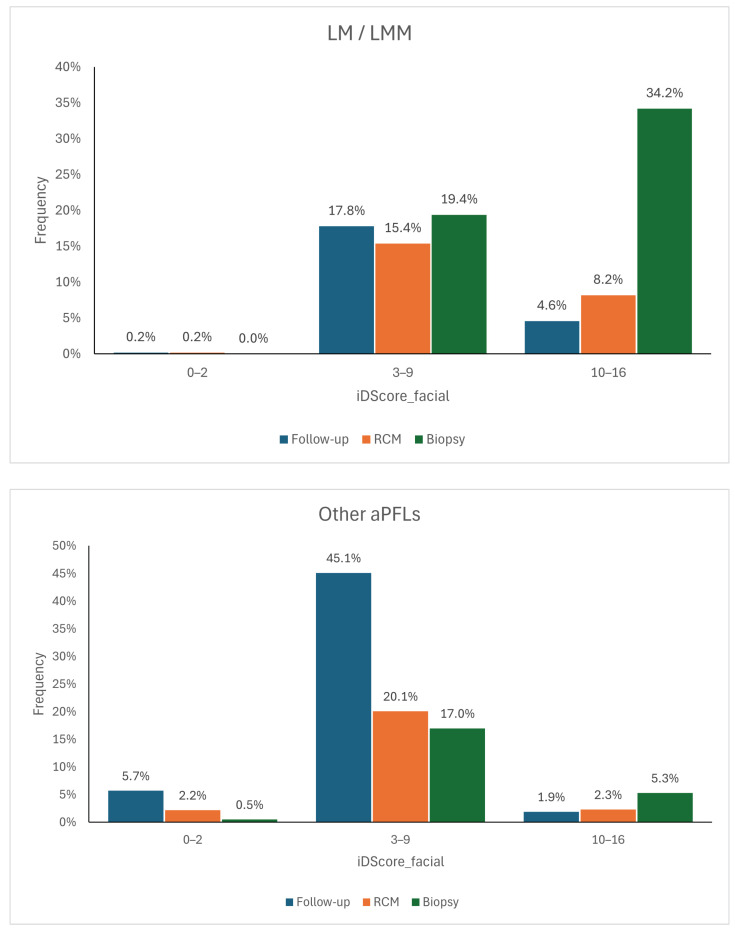
Management distributions according to dermatologists compared to the scores of the model for LM/LMM (**top**) and for other aPFLs (**bottom**) [23].

The logistic regression model and the CNN model have distinct advantages and limitations. The logistic regression model based on iDScore-Facial, grounded in traditional statistical methods, is simpler and easier to integrate into clinical practice. It provides reliable accuracy with a smaller dataset and can easily be understood and implemented by clinicians, as the defined ranges can then be translated into management recommendations, allowing the model to function as a decision support system. If the score falls within the range of 0–2, as cases of malignant lesions are rarely observed within this score range, the lesion can be dismissed or indicated for follow-up. If the score exceeds 10, then the lesion should be biopsied, as lesions with such high scores have a high likelihood of being malignant. Benign lesions with such high scores often exhibit dermoscopic features very similar to those of malignant lesions, so a dermatologist would still opt for their removal. For the intermediate range between 3 and 9, it is not possible to confidently determine whether a lesion is more benign or malignant. Therefore, if the score falls within this range, the recommendation is to conduct further assessments such as follow-ups, examinations with non-invasive diagnostic methods like confocal microscopy, or biopsies. The management suggested by the model was cross-referenced with that directly recommended by the dermatologists and stratified by histology (Figure 7) to quantify the model’s impact on decision-making processes. It can be observed that when using the model, approximately 18% of malignant lesions for which a dermatologist opted for follow-up would instead be assessed with another non-invasive diagnostic tool and therefore potentially recognised as melanomas. Another 5%, on the other hand, would have been biopsied directly. As for benign lesions, the model would have saved approximately 17% of biopsies.

Table 9 shows the performance of both models. For the CNN-based model, only the values for the LM + LMM class are reported so that the performance can be evaluated based on the same binary classification. The comparison of these models highlights the trade-off between simplicity and technological advancement. The CNN model demonstrated higher specificity for melanoma diagnoses, with a diagnostic sensitivity about 23% higher than that of the dermatologists. Its ability to automatically learn and extract meaningful features from dermoscopic images makes it particularly effective in identifying complex patterns. However, the CNN model requires extensive data to achieve clinic-ready accuracy. On the other hand, the logistic regression model is more practical for immediate clinical use. Still, it is also “subjective”, as its training highly relies on the dermatological features identified based on the experience and personal assessments of the physicians. To conclude, the CNN model represents the future directions of AI in dermatology, offering an “objective” way to evaluate dermatological lesions with the potential for greater accuracy as more data become available.

### Limitations

The first major limitation of this study is the scarcity of the dataset of images for training the CNN, which typically requires larger datasets for optimal performance. This shortage of data is mainly because these images depict specific, rare, and hard-to-assess lesions, restricted only to the face area, which currently present a challenge in diagnosis for dermatologists (i.e., the public HAM10000 dataset [27] contains “only” about 750 images related to aPFLs, that is, 37% less than those in the iDScore dataset employed). On the other hand, the LR model incorporates evaluations performed by dermatologists, whose assessments are “learned” by the model . The expertise of the physicians and the number enrolled represent the second major limitation, specifically for the ML model.

## 5. Conclusions

This study provides a significant advancement in the application of AI in dermatoscopy, specifically for significantly enhancing the diagnosis and management of aPFLs. By comparing traditional machine learning approaches, like logistic regression, with advanced deep learning techniques using convolutional neural networks (CNNs), this research demonstrates how AI can enhance the diagnostic accuracy in complex dermatological cases by integrating AI models with clinical data and evaluating different diagnostic approaches, also paving the way for more precise and scalable AI applications in dermatology. Specifically, the logistic regression model offers a practical solution with reliable accuracy, suitable for immediate integration into clinical practice. However, its performance is highly dependent on the experience and evaluations of the dermatologists enrolled in assessing dermoscopic patterns, as they are specific features incorporated into the model, together with patient metadata. The CNN model, despite its higher specificity and precision, faces challenges in integration into clinical settings due to its need for extensive data, requiring further development and resources. The CNN model may still offer improved diagnostic accuracy though, especially in the long term as data resources grow. Equally, it offers an “objective” way to classify aPFLs, as no assessments of the lesions by dermatologists are required to train it. Future research should focus on refining these models, expanding the datasets, and exploring methods to integrate AI-based decision systems into routine dermatological practice. These contributions push the boundaries of AI in medical imaging, showing its critical role in improving patient management and outcomes in dermatology, freeing up physicians’ time and allowing them to focus on more complex cases and critical patient care.

## Figures and Tables

**Table 1:** Distribution of 1197 dermoscopic images collected.

Diagnosis	Distribution of Images
Lentigo Maligna (LM) and Lentigo Maligna Melanoma (LMM)	503 (41.7%)
Pigmented Actinic Keratosis (PAK)	200 (19.3%)
Solar Lentigo (SL)	200 (22.2%)
Atypical Nevi (AN)	194 (10.4%)
Seborrheic Keratosis (SK)	50 (4.0%)
Seborrheic Lichenoid Keratosis (SLK)	50 (2.3%)

**Table 2:** Dermoscopic patterns assessed by dermatologists.

Dermoscopic Pattern	Definition
Hyperpigmented follicular ostia	Fine, irregular semicircles or double circles
Obliterated follicular ostia	Closed follicular openings
Rhomboidal structures	Polygonal lines forming rhomboids
Grey rhomboidal lines	Grey dots/lines arranged in a rhomboidal pattern
Slate-grey dots and globules	Grey dots/globules around follicles
Grey structureless areas	Homogeneous grey areas
Grey pseudo-network	Grey lines forming a pseudo-network
Light brown/dark brown pseudo-network	Brown lines forming a pseudo-network
Fine pigmented brown network	Thin brown lines forming a network
Atypical network	Irregularly arranged network lines
Circle within a circle	Dark circle within a hyperpigmented hair follicle
Irregularly pigmented globules	Dispersed brown/black globules
Dark dots	Black dots within the lesion
Pseudopods	Peripheral projections of pigment

**Table 3:** Parameters set for the CNN training.

Parameter	Value
Initial learning rate	$1\times 10^{-5}$
Maximum epochs	50
Batch size	32
Early stopping	10 epochs
Loss function	Cross-entropy weighted for class frequency
Optimizer	AdamW
Learning rate scheduler	Reduce learning rate on plateau

**Table 4:** Distribution of case ratings, confidence in pattern diagnosis, and management for each diagnosis.

	Atypical Nevus		Lentigo Maligna		Lentigo Maligna Melanoma		Pigmented Actinic Keratosis		Seborrheic Keratosis		Seborrheic Lichenoid Keratosis		Solar Lentigo	
Case rating														
Very easy	11	8.5%	19	14.7%	24	18.6%	23	17.8%	9	7.0%	0	0.00%	43	33.3%
Easy	52	10.1%	115	22.3%	37	7.2%	110	21.4%	26	5.1%	21	4.1%	154	29.9%
Moderate	106	10.2%	306	29.6%	102	9.9%	202	19.5%	44	4.2%	28	2.7%	247	23.9%
Difficult	71	12.5%	177	31.2%	91	16.0%	113	19.9%	19	3.4%	10	1.8%	87	15.3%
Very difficult	27	13.6%	52	26.3%	30	15.2%	35	17.7%	7	3.5%	4	2.0%	43	21.7%
Confidence in diagnosis														
Very confident	24	8.1%	60	20.2%	35	11.8%	47	15.8%	15	5.1%	11	3.7%	105	35.4%
Mildly confident	110	11.6%	249	26.2%	82	8.6%	201	21.1%	54	5.7%	30	3.2%	226	23.7%
Uncertain	72	10.3%	220	31.5%	97	13.9%	139	19.9%	20	2.9%	15	2.2%	135	19.3%
Mildly under-confident	34	13.3%	68	26.7%	42	16.5%	45	17.7%	8	3.1%	2	0.8%	56	22.0%
Not confident	27	11.1%	72	29.6%	28	11.5%	51	21.0%	8	3.3%	5	2.1%	52	21.4%
Management														
Skin biopsy	74	8.8%	314	37.2%	187	22.2%	129	15.3%	28	3.3%	19	2.3%	93	11.0%
Reflectance confocal microscopy	72	12.1%	165	27.7%	64	10.8%	122	20.5%	24	4.0%	20	3.4%	128	21.5%
Close dermoscopic follow-up	121	12.0%	190	18.9%	33	3.3%	232	23.1%	53	5.3%	24	2.4%	353	35.1%

**Table 5:** Accuracy with differently grouped diagnoses.

Diagnosis Categories	Accuracy (%)
Seven diagnoses	42.9
Six diagnoses (Grouped LM with LMM)	48.7
Four diagnoses (Grouped LM with LMM and SL with SLK and SK)	55.8
Two diagnoses (malignant vs. benign)	71.2

**Table 6:** Integer score (iDScore-Facial) of the model for a differential diagnosis of LM/LMM from the other benign facial lesions.

Variable	Coefficient
Maximum diameter ≥ 8 cm	+3
Age ≥ 70 years	+2
Male sex	+1
Presence of rhomboidal structures	+2
Presence of obliterated follicular openings	+2
Presence of a target-like pattern	+2
Presence of hyperpigmented follicular openings	+1
Absence of diffuse opaque yellow/brown pigmentation	+1
Absence of light brown fingerprint-like structures/areas	+1
Absence of red structures and lines	+1
Total score	0–16

**Table 7:** Distribution of LM/LMM and benign cases of the iDScore-Facial model on the testing set for the three identified ranges.

Range	LM/LMM	Benign
Very low (0–2)	0%	19.2%
Intermediate (3–9)	73.3%	73.4%
Very high (10–16)	26.7%	7.3%

**Table 8:** Sensitivity of the CNN model on the testing set compared to the sensitivity of the dermatologists.

Class	Sensitivity (%) of the CNN Model	Sensitivity (%) of the Dermatologists
AN	27.3	48.0
PAK	42.9	42.0
SK	50.0	41.7
SL	50.0	50.0
SLK	100.0	67.9
LM + LMM	78.7	55.5

**Table 9:** Comparison of the sensitivity, specificity, and precision of the CNN and LR models. For the CNN-based model, only the values for the LM + LMM class are reported.

Model	Sensitivity (%)	Specificity (%)	Precision (%)
LR	100.0%	33.9	39.1
CNN	78.7	79.7	75.5

## Data Availability

The data presented in this study are available on request from the corresponding author.

## Abbreviations

The following abbreviations are used in this manuscript:

AI	Artificial Intelligence
AN	Atypical Nevi
AUROC	Area Under the Receiver Operating Characteristic
aPFLs	Atypical Pigmented Facial Lesions
CNN	Convolutional Neural Network
DL	Deep Learning
IARC	International Agency for Research on Cancer
iDScore	Integrated Dermoscopy Score
ISIC	International Skin Imaging Collaboration
LM	Lentigo Maligna
LMM	Lentigo Maligna Melanoma
LR	Logistic Regression
ML	Machine Learning
MLLM	Multimodal Large Language Model
PAK	Pigmented Actinic Keratosis
ROC	Receiver Operating Characteristic
SK	Seborrheic Keratosis
SL	Solar Lentigo
SLK	Seborrheic Lichenoid Keratosis
UV	Ultraviolet
ViT	Vision Transformer
